# Relationship between Outerbridge Scale and Chondropathy Femorotibial Joint in Relation to Gender and Age—The Use of 1.5T and 3.0T MRI Scanners

**DOI:** 10.3390/medicina58111634

**Published:** 2022-11-12

**Authors:** Dominik Sieroń, Izabella Jabłońska, Paweł Niemiec, Dawid Lukoszek, Karol Szyluk, Ivan Platzek, Hugo Meusburger, Georgios Delimpasis, Andreas Christe

**Affiliations:** 1 Department of Radiology SLS, Inselgroup, Bern University Hospital, University of Bern, Freiburgstrasse 10, 3010 Bern, Switzerland; 2 Recreation and Treatment Center “Glinik” 1, Wysowa-Zdrój 101 str, 38-316 Wysowa-Zdrój, Poland; 3 Department of Biochemistry and Medical Genetics, School of Health Sciences in Katowice, Medical University of Silesia in Katowice, Medyków 18 str, 40-752 Katowice, Poland; 4 Dawid Lukoszek Physiotherapy Osteopathy, 42-690 Hanusek, Poland; 5 Department of Physiotherapy, Faculty of Health Sciences in Katowice, Medical University of Silesia in Katowice, 40-752 Katowice, Poland; 6 Department of Orthopaedic and Trauma Surgery, District Hospital of Orthopaedics and Trauma Surgery, Bytomska 62 str, 41-940 Piekary Śląskie, Poland; 7 Department of Radiology, Dresden University Hospital, Fetscherstr. 74, 01307 Dresden, Germany

**Keywords:** femorotibial joint, chondromalacia, aging, body mass index, magnetic resonance imaging

## Abstract

*Background and Objective*: Magnetic resonance imaging (MRI) enables the effective evaluation of chondromalacia of the knee joint. Cartilage disease is affected by many factors, including gender, age, and body mass index (BMI). The aim of this study was to check the relationship between the severity of chondromalacia of the femoro-tibial joint and age, gender, and BMI assessed with 1.5T and 3.0T MRI scanners. *Materials and Methods*: The cross-observational study included 324 patients—159 (49%) females and 165 (51%) males aged 8–87 (45.1 ± 20.9). The BMI of study group was between 14.3 and 47.3 (27.7 ± 5.02). 1.5T and 3.0T MRI scanners were used in the study. The articular cartilage of the knee joint was assessed using the Outerbridge scale. *Results*: The age of the patients showed a significant correlation with Outerbrige for each compartment of the femorotibial joint (Spearman’s rank correlation rho: 0.69–0.74, *p* < 0.0001). A higher correlation between BMI and Outerbridge was noted in the femur medial (rho = 0.45, *p* < 0.001) and the tibia medial (rho = 0.43, *p* < 0.001) than in the femur lateral (rho = 0.29, *p* < 0.001) and the tibia lateral compartment (rho = 0.34, *p* < 0.001). *Conclusions*: The severity of chondromalacia significantly depends on age and BMI level, regardless of gender.

## 1. Introduction

Chondromalacia is a disease affecting the hyaline cartilage that covers the articular surfaces of bones. It causes the cartilage to soften and often leads to tearing and erosion of the cartilage. The environment and physical stress both have an effect on this cartilage. Degeneration of the cartilage also occurs in response to microtraumatic wear. Repeated activities that cause compressive stress on the joint or increased loads on the joint can lead to chondromalacia [1,2,3]. Aging also affects the hyaline cartilage. As we age the number of chondrocytes in cartilage decreases, which correlates with a decrease in the number of proteoglycans produced. This reduction leads to a decrease in the water content of cartilage. A loss of elastic properties develops in the cartilage due to the gradual loss of collagen fibril cross-linking, which also occurs with age. With age, the superficial zone is the first to be damaged [4].

The Outerbridge scale is most commonly used to assess chondromalacia. This classification allows us to categorize cartilage degeneration into four degrees of advancement. It is common to find several degenerative processes at varying levels of severity in the same knee joint [5,6]. MRI is an effective and non-invasive method for evaluating the articular cartilage of the knee joint. This method can be used to detect and monitor degenerative changes that may lead to osteoarthritis [7]. Typical MRI scans (PD and fat-suppressed T2-weighted) can assess the characteristics of cartilage pathology [8]. Together with existing grading scales that assess articular cartilage, MRI can be considered a highly accurate and non-invasive cartilage diagnostic tool [9], with an accuracy of up to 91% [10].

Obesity has a direct correlation with the degeneration of the osteo-articular system. Being overweight causes increased pressure on the articular surfaces of small and large joints, which results in faster wear of the articular surfaces, including cartilage, and its accelerated degeneration [11].

Osteoarthritis (OA) is the most common form of arthritis, affecting approximately 90 million adults (36.8% of the adult population) in the United States alone [12] and hundreds of millions of people worldwide. The disease primarily affects the articular hyaline cartilage in stressed joints, such as the knee joint. Other tissues such as the synovial membrane of the joint capsule and subchondral bone are also affected and contribute to disease progression. As the disease advances, severe cartilage degeneration, joint space narrowing, subchondral bone thickening, osteophyte [13] or bone spur formation, and joint inflammation with associated swelling and pain occur [14]. Increased risk factors for OA are numerous and include obesity, being of the female gender, age, congenital structural defects of the joint, and acute joint trauma [15]. Scientific evidence from systematic reviews show that the progression of knee degenerative changes increases with age and excess weight. In addition, studies indicate that BMI is a significant indicator of the degeneration of articular cartilage in individual compartments of the same knee [16,17,18]. Furthermore, we were interested in the normal range of chondromalacia depending on age, gender and BMI using the Outerbridge classification as quantification of cartilage degeneration. Age and BMI could be used as a direct replacement for the Outerbridge score without the need to perform an MRI.

Due to the large amount of research on patellofemoral chondromalacia, we wanted to improve our understanding of femorotibial cartilage degeneration and check its characteristics. The aim of the study was to investigate the correlation between the severity of chondromalacia of the femorotibial joints and BMI, broken down into categories based on age and gender. Additionally, the differences between using a 1.5T and a 3.0T MRI scanner in the assessment of the knee cartilage were checked.

## 2. Materials and Methods

### 2.1. Study Design

The IRB (Institutional Review Board) states that the presented retrospective studies containing irreversibly anonymized data in our institution do not require the approval of the bioethics committee. In the current observational cross-sectional study, we analyzed the effect of BMI and demographic variables (including age and gender of patients) on the severity of femorotibial joint chondromalacia in consecutive incoming patients undergoing evaluations of knee joint lesions from 2018 to 2019. Patients were recruited from community and clinical hospitals as well as private facilities in Zamość Elblag, Jelenia Góra, and Bielsko Biala (Poland). The medial (femur and tibia separately) and lateral (femur and tibia separately) compartments of the knee joint were evaluated.

The following work is the most recent in a series of scientific publications on chondromalacia of the knee joint. Earlier work included radiological measurements to assess chondromalacia of the knee joint [19]. A paper on chondromalacia of the patellofemoral joint in correlation with BMI depending on age and gender is currently being published. All work was performed based on the same study group and their respective MRI scans.

### 2.2. Description of the Participants

The study group included 324 patients, 159 (47.1%) women and 165 (52.9%) men. A total of 155 (47.8%) patients, including 70 (45.2%) women and 85 (54.8%) men, were examined on the 1.5T scanner, while 169 (52.2%) patients, including 89 (52.7%) women and 80 (47.3%) men, were examined on the 3.0T scanner. Four age classes were defined for the study: <30 years (94 participants); 30–45 years (61 participants); 46–60 years (78 participants); >60 years (91 participants).

### 2.3. Inclusion and Exclusion Criteria

Inclusion criteria: patients with pain or suspected osteoarthritis or post-traumatic lesions who on the order of an orthopedist, surgeon, or physiotherapist submitted for an MRI. Several patients also submitted for a private scan at their own request due to complaints of joint pain.

Exclusion criteria: previous knee surgery or chronic post-traumatic changes of the knee.

### 2.4. Evaluation of Cartilage Chondromalacia

To assess cartilage chondromalacia, we used the 5-level Outerbridge classification (Table 1) using fat-saturated proton density sequences—a modified classification for arthroscopic cartilage assessment [16,17,18,19]. Three board-certified radiologists with 12, 25 and 32 years of experience in musculoskeletal imaging classified the MR images according to the Outerbridge score. Cases were randomly and equally distributed among the radiologists. The MRI scans reported by the radiologists did not overlap. For this reason, consistency between radiologists was not assessed.

### 2.5. Image Acquisition

The study was evaluated on an iMac pro (Apple, Cupertino, CA, USA) using the FDA approved OsiriX MD software (version 11.0, Pixmeo SARL, Bernex, Switzerland). MRIs were performed on 3.0T scanners (Ingenia 3.0T, Philips, Amsterdam, The Netherlands) and on 1.5T GE scanners (SIGNA, GE, Milwaukee, WI, USA) GE at different facilities located in clinical hospitals and private facilities in Zamość, Elbląg, Jelenia Góra, and Bielsko-Biala. All MRI studies were irreversibly anonymized [16].

The following diagnostic sequence protocol was used in the study: axial, sagittal and coronal PD FS, sagittal and coronal T1 (all with a slice thickness of 3 mm), 3D high-resolution PD FS with a slice thickness from 0.8 to 1 mm. The same protocol we used in the earlier publication [19].

### 2.6. Statistical Analysis

A Chi-square (χ^2^) test of independence was used to analyze the difference of chondromalacia in the various age, sex and MRI-unit groups. The correlation between the age subgroups and Outerbridge scale chondromalacia lesion scores, the C contingency co-efficient, was calculated, and the R-Spearman rank correlation was checked. The comparison of correlation coefficients was performed using the z statistic.

Subsequently, the correlation between BMI level and Outerbridge chondromalacia lesion scores was tested using the Kruskal–Wallis test, ANOVA, post-hoc analysis (Conover test), the trend for mean values (Me) of BMI level in each Outerbridge scale grade, the Jockheere–Terpstra trend test, and the R-Spearman (rho). The analysis was performed for the whole group, 1.5T and 3.0T, for females and males—separately for 1.5T and 3.0T—and a pooled analysis of Outerbridge score 0/1/2 vs. 3/4 was performed. The continuity coefficient was used as a measure of the relationship between the age subgroup and Outerbridge scale grade. The use of the χ^2^ test provided the basis for the calculation of the C coefficient. Our table had dimensions of 4 × 4; in this case Cmax = 0.866. To compare the impact of the different variables (age, BMI, sex and MRI scanner type) on the Outerbridge score, a logistic regression model was used with the dependent dichotomous variable being no or slight degeneration (Outerbridge score 0/1/2) and severe degeneration (Outerbridge score 3/4).

## 3. Results

### 3.1. Age

**Figure 1:**
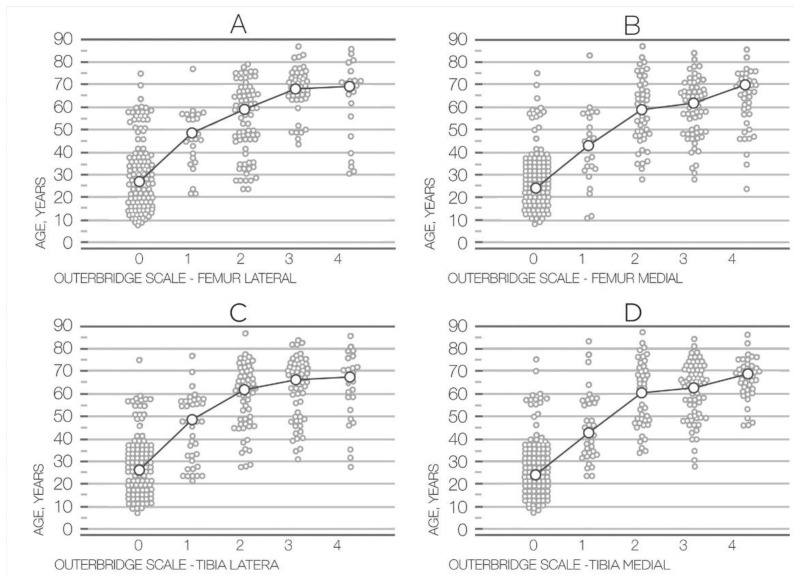
Relationship between the Outerbridge scale and age for femur lateral (**A**), femur medial (**B**), tibia lateral (**C**), and tibia medial (**D**).

Age demonstrated significant correlation coefficients (Spearman rank correlation, *p* < 0.001) with Outerbridge for the femur lateral (rho = 0.69), tibia lateral (rho = 0.71), femur medial (0.72), tibia medial (rho = 0.74) (Table 2, Figure 1).

### 3.2. BMI

The mean BMI for the entire study group was 27.7; SD: 5.02. The highest values of BMI were noted in the patients diagnosed with Outerbridge Scale 2 for the tibia lateral and Outerbridge Scale 4 for the femur medial and tibia medial. For the individual parameters of the Outerbridge scale, the mean BMI is presented in Table 3.

#### 3.2.1. Femur Lateral

**Figure 2:**
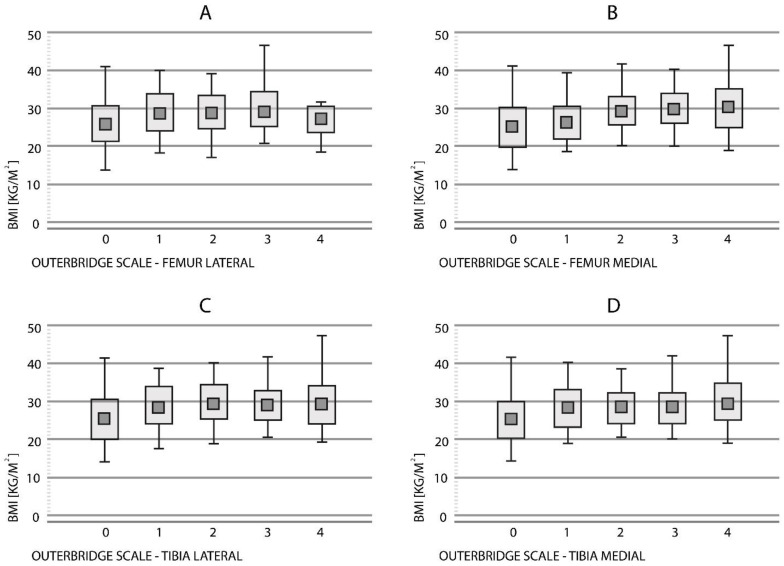
Relationship between BMI and Outerbridge scores using both scanners (1.5T and 3.0T) in the entire study group for femur lateral (**A**), femur medial (**B**), tibia lateral (**C**), and tibia medial (**D**).

A statistically significant positive correlation was found between BMI level and Outerbridge parameter score (rho = 0.29, *p* < 0.001) (Figure 2A).

There was a statistically significant difference in BMI level according to the Outerbridge parameter score (KW, *p* < 0.001) in the entire study group of patients (scanner 1.5T and 3.0T).

For the femur lateral, there were no significant statistical differences between the Outerbridge subgroups (1 and 2 vs. 3 and 4) in terms of the BMI level (U-test, *p* = 0.39).

The grade 0 Outerbridge parameter score showed a statistically significant lower mean BMI level compared to grades 1, 2 and 3 (*p* < 0.05).

#### 3.2.2. Femur Medial

There was a statistically significant positive correlation between the BMI level and Outerbridge parameter score (rho = 0.45, *p* < 0.001) for both scanners (Figure 2B).

There was a statistically significant difference in BMI level between the Outerbridge scores (*p* < 0.001) in the entire study group.

For the femur medial, there were significant statistical differences between the Outerbridge subgroups (1 and 2 vs. 3 and 4) in terms of the BMI level (*p* = 0.01).

The grade 0 Outerbridge parameter score showed a statistically significant lower mean BMI level compared to grades 2, 3 and 4 (*p* < 0.05).

#### 3.2.3. Tibia Lateral

A significant correlation was found between the BMI level and Outerbridge parameter score (rho = 0.34, *p* < 0.001) for both scanners (Figure 2C).

The parameters’ Outerbridge scores significantly differed statistically in terms of the BMI (*p* = 0.0001) for both scanners.

For the tibia lateral, no significant differences were found between the Outerbridge subgroups (1 and 2 vs. 3 and 4) in terms of the BMI level (*p* = 0.72)

The grade 0 Outerbridge parameter score showed a statistically significant lower mean BMI level compared to the level of the rest of the parameters (*p* < 0.05).

#### 3.2.4. Tibia Medial

There was a statistically significant relationship between the BMI level and Outerbridge parameter score (rho = 0.42, *p* < 0.001) at this location (Figure 2D).

The Outerbridge score parameters were significantly statistically different in terms of the BMI level (*p* < 0.001) at this location.

The Outerbridge subgroups (1 and 2 vs. 3 and 4) differed significantly in terms of the BMI level for the tibia medial (*p* = 0.017).

The grade 0 Outerbridge parameter score showed a statistically significant lower mean BMI level compared to the level of the rest of the parameters (*p* < 0.05).

### 3.3. Gender

There were no significant differences between women and men in the Outerbridge assessment for each knee joint compartment in each age subgroup (test χ2, *p* > 0.05).

#### 3.3.1. Women

For women, age demonstrated the following correlation coefficients (Spearman rank correlation, *p* < 0.001) with Outerbridge: for the femur lateral (rho = 0.72), the tibia lateral (rho = 0.71), the femur medial (rho = 0.70), and the tibia medial (rho = 0.70).

#### 3.3.2. Men

For men, age demonstrated the following correlation coefficients (Spearman rank correlation, *p* < 0.001) with Outerbridge: for the femur lateral (rho = 0.66), the tibia lateral (rho = 0.71), the femur medial (rho = 0.74), and the tibia medial (rho = 0.77).

### 3.4. Type of Scanners (1.5T vs. 3.0T)

The demographics of the two groups were as follows: A total of 70 (45.2%) females and 85 (54.8%) males were examined on the 1.5T scanner, while 89 (52.7%) females and 80 (47.3%) males were examined on the 3.0T scanner. The average age of the 1.5T and 3.0T group was 43.8 ± 18.7 and 46.9 ± 23.5 years, while the mean BMI of the 1.5T and 3.0T group added up to 28.2 ± 5.0 and 27.1 ± 5.2.

Significant differences were found between the scanner type (1.5T vs. 3.0T) in the Outerbridge assessment for the femur lateral (χ2, *p* = 0.002), tibia lateral (χ2, *p* = 0.006), femur medial (χ2, *p* = 0.034), and tibia medial (χ2, *p* = 0.007) in the entire study group:

For the 1.5T scanner, the Outerbridge scale scores were in general higher than for the 3.0T scanner (Table 4).

When all joint compartments were evaluated together, the correlation coefficient of the Outerbridge and BMI at 1.5T/3.0T was 0.3997/0.3303 (*p* = 0.1314) and the correlation of the Outerbridge and age at 1.5T/3.0T was 0.687/0.777 (*p* < 0.001).

### 3.5. Logistic Regression of Age, BMI, Sex and MRI Scanner Type on Outerbridge Score

Age, BMI and MRI scanner type played a significant role in predicting the severity of knee degeneration (Outerbridge 3 and 4) with the following logistic regression formula:

Logit (Outerbridge 3/4) = 0.0888 × A + 0.0864 × B + 0.0604 × M − 0.717 × T − 6.58(1)

A (age), B (BMI), M (male patient) and T (1.5 Tesla MRI) demonstrated odd ratios of 1.093 (95% CI: 1.073 to 1.113), 1.090 (95% CI: 1.026 to 1.159), 1.062 (95% CI: 0.578 to 1.953) and 0.488 (95% CI: 0.265 to 0.901), respectively. According to the *p*-value, the influence was strongest for the age (*p* < 0.001), followed by BMI (*p* = 0.005) and MRI type (*p* = 0.022); sex did not have a significant impact on degeneration prediction (*p* = 0.85).

## 4. Discussion

The results showed a significant correlation between the Outerbridge chondromalacia score and BMI and age. A similar correlation was noted in a study by Matada, who additionally noted that a significant increase in larger knee cartilage lesions occurs in individuals > 50 years and BMI > 25 [24]. Chondromalacia changes are noted when assessing structural changes in osteoarthritis [21]. Risk factors for symptomatic knee joint changes due to osteoarthritis include obesity and age [25]. The relationship between BMI and knee OA is mainly linear [26].

### 4.1. Gender

Previous studies have noted differences in the extent of cartilage loss of the femorotibial joint between men and women, with greater degeneration observed in women than in men [27,28,29]. Despite these reports lacking clarity, the hypothesized mechanism for the significant progression of knee cartilage disorders involves biomechanical and hormonal factors that distinguish women from men [28]. In women, the progression of degenerative changes of the knee joint significantly increases after the age of 50 in the postmenopausal period [27]. A cross-sectional study found that women taking hormone therapy in the postmenopausal period exhibit greater cartilage volume [30]. Possible biomechanical differences in gait and the knee joint in women may also accelerate the development of OA [31,32]. Our study showed no significant difference in the Outerbridge scores between women and men. Additionally, the correlation of Outerbridge and age/BMI was the same between both genders.

### 4.2. Aging

The development of knee osteoarthritis is closely related to aging [33,34]. The catabolic–anabolic imbalance of cartilage causes matrix destruction through excessive oxidation of antioxidant systems in chondrocytes, including glutathione and peroxiredoxins [35]. Joint components that undergo changes due to aging also contribute to the degeneration of hyaline cartilage. Osteoporosis or the weakening of the quadriceps muscle of the thigh leads to the dysfunction of the femorotibial joint, increasing the maximum stresses on the cartilage [36,37,38]. In the analysis of this study, a significant relationship was found between the presence of greater chondromolytic changes and increasing age for this group.

### 4.3. BMI

For the entire group, a positive correlation was found between BMI and Outerbridge scores. A higher correlation between BMI and Outerbridge was found in the medial compartments, femur medial (rho = 0.45) and tibia medial (rho = 0.42), than the lateral compartments, femur lateral (rho = 0.29) and tibia lateral (rho = 0.34). It should be noted that obesity is not only associated with osteoarthritic changes of the knee joint in the mechanical but also in the metabolic background [39,40,41]. Through research, adipokines, leptin, and resistin, which have endocrine functions in adipose tissue, have been identified [38]. In vivo, findings have indicated that there is a detrimental effect on chondrocyte proliferation as well as the initiation of extracellular matrix metalloproteinase expression, resulting in reduced cartilage volume [42].

### 4.4. MRI and the Assessment of Cartilage Chondromalacia

A comparison of knee joint images from the same patients on the 1.5T and 3.0T apparatus in Wang’s study, followed by arthroscopy, concluded that the visualization of anatomical structures and the confidence in making diagnoses of cartilage lesions are both improved when using 3.0T scanners. The reliability (88.2% vs. 86.4%) and sensitivity (51.3% vs. 42.9%) of the 3.0T device was improved compared with the 1.5T. The correct assessment of cartilage damage differed in favor of the 3.0T apparatus (51.3 vs. 42.9%) [43]. A study by Mandell, who used a similar methodology to Wang in examining a larger group, inferred no significant differences between the 1.5T and 3.0T [44]. In contrast, a systematic review and meta-analysis concluded that the 3.0T scanners were significantly more reliable for imaging articular cartilage [10] with age-related degeneration. In our study, the 1.5T scanner scored higher Outerbridge levels in all compartments compared to the 3.0T unit, but the correlation coefficients of Outerbridge and BMI did not differ, and the correlation coefficient of Outerbridge and age was even significantly higher than with the 3.0T unit. Therefore, one explanation for the higher Outerbridge scores of the1.5T scanner could be a possible geographical inclusion inhomogeneity. From our results, it seems that both the 1.5T and the 3.0T apparatuses are effective methods of evaluating Outerbridge cartilage according to the BMI level. On the other hand, the 3.0T apparatus may have an advantage in terms of age correlation.

### 4.5. Limitations

One major drawback of our study is the lack of a comparison of the same group of subjects using the 1.5T and 3.0T apparatus. The lack of a characterization of the group in terms of additional injuries such as ACL, meniscus, fat pad, and other factors such as physical activity or quadriceps thigh muscle strength made it impossible to test for differences in Outerbridge scores. The most important limitation of the study is the BMI used, which shows low sensitivity in obesity research and non-specificity in chondromalacia studies [45].

## 5. Conclusions

Assessing the degree of cartilage degenerative changes with 1.5T and 3.0T MRI is an effective form of lesion classification using the Outerbridge scale.

A positive correlation was observed between the degree of chondromalacia in both compartments of the femorotibial joint and the body mass index and age of the subject.

No differences were noted between men and women in the assessment of articular cartilage.

Our suggestion for further research is a longitudinal follow-up to evaluate the effect of the duration of obesity during life on cartilage changes and to see which apparatus, the 1.5T or 3.0T, is more effective in monitoring patients with OA. In order to provide a complete answer to the question of whether age and BMI can be used as a variable in the Outerbridge scale without the use of MRI, a predictive model study with multivariable analysis should be performed, taking into account many factors causing a predisposition to chondropathy of the femorotibial joint.

## Figures and Tables

**Table 1:** Outerbridge classification [19,20,21,22,23].

Grade	Macroscopy	MRI
Grade 0	Normal cartilage	Normal cartilage
Grade 1	Rough surface; chondral softening; focal thickening	Inhomogeneous; high signal; surface intact; cartilage swelling
Grade 2	Irregular surface defects; <50% of cartilage thickness	Superficial ulceration, fissuring, fibrillation; <50% of cartilage thickness
Grade 3	Loss of >50% cartilage thickness	Ulceration fissuring, fibrillation; >50% of depth of cartilage
Grade 4	Cartilage loss	Full thickness chondral wear with exposure of subchondral bone

**Table 2:** Relationship between the Outerbridge scale and age for particular compartments of the femorotibial joint.

Correlated Variables	N	R_s_ Spearman	T (N-2)	*p*
Femur Lateral & Age	324	0.69	16.9308	<0.001
Femur Medial & Age	324	0.72	18.8962	<0.001
Tibia Lateral & Age	324	0.71	18.3634	<0.001
Tibia Medial & Age	324	0.74	20.0209	<0.001

**Table 3:** BMI for Outerbridge scale.

Outerbridge	BMI (Mean ± SD)			
	Femur Lateral	Tibia Lateral	Femur Medial	Tibia Medial
Grade 0	26.0 ± 4.88	25.7 ± 4.92	25.3 ± 4.47	25.4 ± 4.55
Grade 1	29.1 ± 5.31	28.8 ± 4.97	26.4 ± 4.17	28.2 ± 4.71
Grade 2	29.4 ± 4.49	29.6 ± 4.54	29.4 ± 3.85	28.3 ± 3.97
Grade 3	29.6 ± 5.00	28.8 ± 4.06	29.9 ± 4.90	28.3 ± 3.97
Grade 4	27.0 ± 3.54	29.4 ± 5.33	30.3 ± 5.01	29.6 ± 4.78

**Table 4:** Outerbridge Scale for each compartment of femorotibial joint.

Outerbridge Scale		
	*1.5T MRI scanner*	*3.0T MRI Scanner*
Femur Lateral	1.387 ± 1.306	1.225 ± 1.400
Tibia Lateral	1.581 ± 1.391	1.207 ± 1.379
Femur Medial	1.794 ± 1.528	1.367 ± 1.503
Tibia Medial	1.709 ± 1.503	1.260 ± 1.485.

## Data Availability

Data are available upon special request.
